# An Approach to the Consumption of Smoked Paprika in Spain and Its Impact on the Intake of Polycyclic Aromatic Hydrocarbons

**DOI:** 10.3390/foods10050973

**Published:** 2021-04-29

**Authors:** José M. Coleto, Alberto Martín, Andrés Horrillo, Francisco J. Mesías, Rocío Velázquez

**Affiliations:** 1 Department of Agri-forest Engineering, University of Extremadura, 06006 Badajoz, Spain; jmcoleto@unex.es (J.M.C.); rvotero@unex.es (R.V.); 2 Department of Animal Production and Food Science, University of Extremadura, 06006 Badajoz, Spain; amartin@unex.es; 3 Department of Economics, University of Extremadura, 06006 Badajoz, Spain; andreshg@unex.es

**Keywords:** smoked paprika, consumption, Protected Designation of Origin (PDO), polycyclic aromatic hydrocarbons, manual production

## Abstract

“Pimentón de La Vera” smoked paprika is a traditional kind of smoked paprika, the production of which is regulated by a protected designation of origin. The traditional drying/smoking process provides the “Pimentón de La Vera” smoked paprika with a peculiar flavour which has gained acceptance in multiple markets. However, this process also gives rise to non-desirable substances, such as polycyclic aromatic hydrocarbons (PAHs). This paper attempts to ascertain the consumption levels of smoked paprika per person in Spain in order to establish the intake of PAHs derived from this food spice. With this purpose in mind, a research study was carried out using questionnaires in three different smoked paprika consumption scenarios: food companies, households and restaurants. The results from this research proved that the average consumption of smoked paprika per person per year in Spain is 139 g. Overall, the intake of PAHs derived from smoked paprika was proven to represent a negligible fraction of the total intake, with this ingredient being far behind the PAH contribution represented by other food products. These results could help consolidate the smoked paprika production sector by providing evidence of the scarce contribution of smoked paprika to PAH intake and justifying the traditional production with smoke drying, which is the differentiating quality trait of this spice.

## 1. Introduction

“Pimentón de La Vera” smoked paprika is a traditional type of smoked paprika produced in four counties of the north of Extremadura (southwest of Spain), the peculiarities of which have granted it protected designation of origin (PDO) recognition [1]. The pepper was brought to Spain from America by Christopher Columbus in the 15th century, and Spain began to spread it as a crop in the 16th century, because it was used as a replacement for the expensive black pepper (*Piper nigrum* L.) from Asia and because it was very easy to grow in Spanish soil and weather conditions [2]. The paprika sector in Extremadura is relevant from the economic and social point of view, as it is a source of wealth for farmers, manufacturers and the labour force required, mainly during the harvesting stage. The annual production of peppers for paprika in the region is 4.013 tons, and the economic value received by Extremaduran producers amounts to EUR 12.31 million. [3].

Although peppers were mainly used as vegetables, two very specific areas of Spain, i.e., the La Vera region in the north of Extremadura and La Huerta region in Murcia (southeast of Spain), soon began to specialise in the production of its spice, paprika (dry and ground pepper) [2]. The differences in the weather conditions in the autumn—the season in which peppers are collected and dried—between these two regions determined the drying process used in each region. Thus, the warm autumn in Murcia allowed the peppers to be dried under the sun, whereas the very humid autumn in La Vera led to the adoption of the smoke-drying technique burning holm oak or common oak wood. This peculiar drying process, together with the origin and the varieties used (*Capsicum annuum* L., Jaranda, Jariza, Jeromín and Bola varieties), gives rise to unique sensorial and compositional traits. Among these, the smoked flavour and aroma can be highlighted, as well as the stability of the intense red colour due to the high content of carotenoid pigments and to the traditional drying process. [4,5,6,7].

The production process of the “Pimentón de La Vera” smoked paprika starts with the collection of the peppers, when the fruit is ripe. The smoke-drying process follows tradition and uses two-floor drying houses with the fire burning on the ground floor and the peppers stored on the first floor. The drying process is slow, taking between 10 to 15 days, during which the farmer turns the product manually every day so that the fruit dries evenly.

However, this drying/smoke-drying process that is responsible for the peculiar flavour of “Pimentón de La Vera” smoked paprika and its acceptance in the market also gives rise to undesirable substances, such as polycyclic aromatic hydrocarbons (PAHs). Various studies have proven that the habitual processes used in the production of food products, such as drying and smoke-drying, can produce PAHs [8,9]. Therefore, and with the objective of protecting public health, the maximum levels of these contaminants in foods that have undergone smoke-drying and air-drying processes were set, as they can cause high levels of contamination [10].

Initially, benzo(a)pyrene (B(a)P) was used as an indicator of the presence and effects of PAHs in food [11]. However, at a later stage, the European Food Safety Authority (EFSA) [12] recommended the use of the four PAHs (PAH4): benz(a)anthracene (B(a)A), chrysene (CHR), benzo(b)fluoranthene (B(b)F) and benzo(a)pyrene (B(a)P), as they proved to be better indicators of the levels of PAHs in food.

The law establishes that since 1st of April 2016, dry cooking herbs and spices that are sold in the EU markets cannot exceed the maximum level of 10 μg/kg for B(a)P or 50 μg/kg for PAH4 [13,14]. Nevertheless, since the spice obtained from smoked *Capsicum* fruits is only used in small amounts per person, it is exempt from complying with the minimum levels required for smoked products in order to be commercialised [13]. In terms of the content of PAHs in smoked meat and meat products produced using the traditional methods in Spain and intended for consumption in the national territory, these products cannot exceed 5 μg/kg of B(a)P and 30 μg/kg of PAH4 [15,16].

There are currently very few references on PAH content in dry red peppers, smoked paprika and processed products containing this spice. However, the authors of [17] carried out a study with three dried red pepper samples from Japanese markets, which stated that the B(a)P content was 4.5 μg/kg and the PAH4 content was 35.5 μg/kg.

On the other hand, the authors of [18] analysed the presence of PAHs in sweet paprika and chili peppers from Brazil and China, and they found an average concentration of B(a)P and PAH4 of 1.09 and 8.04 μg/kg, respectively. Another research study [19] found average values of 67 μg/kg B(a)P and 1776 μg/kg PAH4 in samples of smoked paprika from countries other than Spain. 

In terms of meat products, the authors of [20] analysed the PAH content in two traditionally smoked pork sausages from Spain containing smoked paprika, i.e., “Chorizo gallego” and “Chorizo de cebolla”. The mean B(a)P levels were 0.72 and 1.17 μg/kg, respectively, with PAH4 levels being also well below the maximum authorized levels of 30.0 μg/kg of PAH4 [15,16]. Similar results were found in other Spanish sausages [21].

Another study [22] analysed samples of “Pimentón de La Vera” smoked paprika and Spanish “chorizo”. The average B(a)P concentration was 104 μg/kg, and the average PAH4 concentration was 1602 μg/kg in smoked paprika. In terms of the analysed “chorizo” samples, which are processed products containing smoked paprika and dried using the traditional methods with wood smoke, the average B(a)P and PAH4 contents were 26 and 363 μg/kg, respectively.

However, increasing consumer concern for the impact of food on health may cause future restrictions in terms of the presence of PAHs. This, together with the inherent variability of the traditional “Pimentón de La Vera” smoked paprika production process, has raised concerns in the production sector of the production areas, since the industrial activities connected to growing peppers and the production of smoked paprika provide employment for a significant proportion of the population and create wealth in the region.

This is why “Pimentón de La Vera” smoked paprika producers are working on ways to reduce PAH content without reducing the special sensorial properties, the antioxidant activity, the colour and colour stability of smoke-dried peppers [7,23,24]. “Pimentón de La Vera” smoked paprika is specially used as a spice to add colour, flavour and aroma to dry-cured pork sausages, and to many culinary recipes [25]. However, there are no data relating to the amount of smoked paprika being consumed per person in Spain, precisely because it is used in small amounts and is often classified together with other spices and flavourings in official statistics [26]. Consequently, it is of great interest for the sector to have accurate consumption data, which requires an analysis of household and restaurant usage, since smoked paprika is mainly consumed in these two scenarios. Apart from the organoleptic, sensorial and health-related properties of “Pimentón de La Vera” smoked paprika, various clinical studies published by [27,28] describe the health effects that smoked paprika consumption has in healthy young people in association with its strong antioxidant action. 

In view of the above, the purpose of this paper is to ascertain the per capita consumption of smoked paprika in Spain and use the resulting values to determine the intake of smoked paprika-sourced PAHs in Spanish consumers. In order to do so, three studies were undertaken: one with Iberian dry-cured pork sausage production companies in order to ascertain the average percentage of smoked paprika that is used in the most significant processed meats. Subsequently, the use of smoked paprika in households and restaurants was analysed, estimating the consumption of dry-cured pork sausages in both environments and the use of smoked paprika in cooking. 

The results of this study provide a tool to determine the contribution of “Pimentón de la Vera” smoked paprika to the daily intake of PAHs, related to the traditional processing system, which is the distinctive mark of this spice.

## 2. Materials and Methods

### 2.1. Area of Study

The data were collected through an adaptation of the procedure used by the Consumer and Food Distribution Observatory of the Ministry of Agriculture, Fisheries and Food of the Spanish Government [29], in order to adjust it to the specific features of this study. Thus, personal interviews were carried out instead of telephone interviews, and the food products under analysis were reduced to industrially processed foods and cooked foods that use smoked paprika.

The geographical scope of the study was the region of Extremadura, as it is a relevant area for the production of smoked paprika and is among the Spanish regions with the highest level of consumption of processed meat products that use smoked paprika in their production (“chorizo” and dry-cured pork loin) [26]. Since the purpose was to ascertain the intake of PAHs, the high level of consumption of dry-cured pork sausages in Extremadura (and the consequent high level of consumption of smoked paprika) allows for the estimated data of consumers of this region to be easily transposed to the rest of the Spanish population. Interviews were directed to the three scenarios of the most significant smoked paprika consumption: consumers, restaurants and companies producing processed meat products using smoked paprika as a flavouring ingredient.

Due to the lack of up-to-date data on the consumption of smoked paprika, the recommendations made by [29] were taken into account in order to calculate the size of the sample, together with a set of pre-tests applied to 28 households and 10 restaurants in order to ascertain their use of smoked paprika and, therefore, be able to estimate the sample error.

The final sample consisted of 200 consumers, 40 restaurants and 10 processed meat production companies, which would provide an estimation of the consumption of smoked paprika with an error level acceptable for these types of studies. The distribution of surveys to consumers and restaurants within the region followed a method by natural geographical areas, where the number of surveys was allocated proportionally to their population.

### 2.2. Questionnaire

#### 2.2.1. Consumer Questionnaire

Households were surveyed taking into account two different perspectives or dimensions. The first dimension was related to the consumption in the household of the various processed food products containing smoked paprika or food with smoked paprika flavouring. The second dimension concerned the number of meals each member of the household consumes on an annual basis either individually or accompanied by other members of the household in restaurants or food establishments.

In the same way, the survey contained a number of introductory questions on the number of members in the household, occupation of the chief earners, age of the individuals responsible for the food shopping, occupation, employment situation and estimated level of income.

#### 2.2.2. Restaurant Questionnaire

In this case, two different dimensions were also established: The first dimension, which is similar in the survey taken by households, was intended to calculate the intake of foods with smoked paprika in their ingredients within a week.

The second dimension was related to the number of meal-equivalents the restaurant served per week. In order to calculate the meal-equivalents, the following rules were established:

Breakfast: This meal is not directly included, as smoked paprika usually has a low presence in this meal, although this intake is indirectly prorated amongst the main meals.

Tapas: every three tapas are counted as a main meal (weighing: 0.33).

Lunch and dinner: these are counted as main meals (weighing: 1.00).

The data on the consumption of smoked paprika in processed food and as a spice and the number of meal-equivalents, both calculated for a specific period of time—a week in this case—enabled us to calculate the average amount of smoked paprika consumed in each meal at the various types of establishments. Given that the household surveys helped us ascertain the number of meals that each member of the household consumes annually outside the home, the individual consumption of smoked paprika outside the home was calculated using the following formula:

Individual consumption outside the home (g year^−1^) = average consumption of meal-equivalents (g) × number of meals year^−1^. (1)

#### 2.2.3. Processed Meat Product Company Questionnaire

In this case, the survey intended to determine the average smoked paprika content in the industrial processed products consumed afterwards by households and restaurants.

The information of interest collected from these companies was related to the percentage of smoked paprika used in their processed products, for which we collected information on the types of processed meat products that are most common in the region (various types of “chorizo” and other dry-cured meats, such as pork loin).

The questionnaires used in this study are included as supplementary files (Tables S1–S3).

### 2.3. Data Collection

As previously mentioned, the surveys adopted a face-to-face format in all instances. For the consumer survey, when the relevant number of surveys was allocated to the various geographical areas, the interviewer would visit these areas and select the consumers randomly by approaching them in public areas, such as town squares or shopping centres. For restaurants and industries, given the smaller population size, the approach was to contact people over the phone using the phone directory, later visiting those who were willing to participate in the research.

To date, this is the most complete study on smoked paprika consumption that has been carried out, since this product, due to its consumption levels, is usually included with other condiments or spices. The surveys were carried out considering one year’s consumption to avoid seasonal fluctuations, and the data obtained correspond to the average of the surveys.

### 2.4. PAH Determination

For the purposes of this study, reference values for polycyclic aromatic hydrocarbons from 144 samples of PDO “Pimentón de La Vera” smoked paprika were taken, which derived from a 4-year study (2015 a 2018) performed at the agrifood laboratory of the Escuela de Ingenierías Agrarias of the University of Extremadura [30], which is a laboratory certified according to the requirements of the UNE-EN-ISO 9001:2015 reference standard. The variability associated with the different processing procedures of smoked paprika were also taken into account (see supplementary files, Table S4). The B(a)A, CHR, B(b)F and B(a)P compounds were extracted using the QuEChERS [31] methods and analysed by HPLC using a Hewlett-Packard 1100 series instrument (Palo Alto, CA, USA) equipped with a fluorescence detector (260ex/420 em) and a SUPELCOSIL™ LC-PAH HPLC Column (Supelco, Inc., Bellefonte, PA, USA). Additional information about the conditions and validation of the method for smoked paprika is showed in Table S5.

The resulting average values of 0.049 mg kg^−1^ of B(a)P and 0.780 mg kg^−1^ of PAH4 served as a basis for the calculation of the annual consumption of PAHs (mg y^−1^) per person derived from the consumption of food containing smoked paprika in Extremadura.

**Figure 1:**
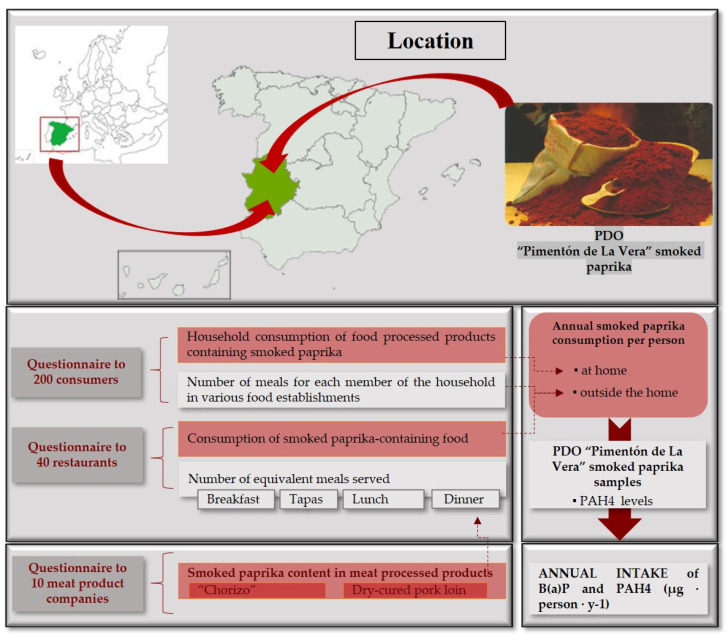
Methodological process. PDO: Protected Designation of Origin.

Figure 1 summarises the methodological process followed in this research.

## 3. Results and Discussion

This section is structured as a sequence, starting with the presentation of the survey results with the purpose of obtaining information on the content of smoked paprika in processed meat products and the total consumption of smoked paprika by consumers derived from the consumption of the aforementioned processed meat products and other food products where smoked paprika is used as a spice.

### 3.1. Estimation of Smoked Paprika Consumption

Table 1 shows the percentage of smoked paprika used in the composition of the main processed meat products produced by the 10 Extremaduran companies that were interviewed.

As can be observed in Table 1, the surveyed companies provided data on each processed meat product with very little differences between them. In the case of “chorizo” and similar products, the average is 1.995% in the mature product that is ready for the market, whereas for dry-cured pork loin, it is 1.001%, which are similar amounts to the ones used traditionally in the manual production of cured meats from Leon (northwest of Spain) [32]. In the northeast of Spain, the amount of smoked paprika employed is slightly higher, as they use specifically 2.5% of “Pimentón de La Vera” smoked paprika in order to produce “chorizos” [33].

Table 2 reveals the results on the consumer consumption of processed meat products containing smoked paprika (“chorizo” and dry-cured pork loin) as well as the use of smoked paprika directly in the making of meals. As mentioned in the methodology section, the consumption data in households, traditional restaurants and fast-food restaurants are reflected.

According to Table 2, the total consumption of smoked paprika is 139 g per person per year. In addition, in Extremadura households, the annual consumption of processed meat products containing smoked paprika per person adds up to 2.86 kg of “chorizo” in its different varieties and presentations (various types of “chorizo”, potato sausage, black pudding and their sliced versions) and 0.95 kg of dry-cured pork loin and its sliced versions. In terms of the smoked paprika purchased in order to use it as a seasoning or in marinades, the average is 56 g per person per year.

The consumption of processed meat products and cooked products containing smoked paprika outside the home depends on the meals the members of the household consume in the various types of food establishments. As previously stated in the Methodology section, apart from the main meals, it also includes the tapas and breakfasts. The total consumption of dry-cured meat products in restaurants is 0.57 kg, the majority of which (0.48 kg) takes place in traditional food establishments.

The total annual consumption of processed cured meat products containing smoked paprika per person is 4.38 kg, which is noticeably higher than the figure of 1.8 kg per person consumed in Spain according to the studies of [26,34]. This figure confirms that the amounts consumed in Extremadura are clearly above the overall figures for Spain, a fact that proves that the choice of the Extremadura region for this study was appropriate.

Table 3 summarizes the total consumption data for smoked paprika per person per year, on the basis of the data collected from the previous table on the smoked paprika content of processed meat products and their direct consumption through its use in cooking.

The conversion of processed meat products and cooked products into their equivalent smoked paprika intake (Table 3) reveals that the average consumption of smoked paprika in Extremadura is 139 g per person per year, the majority of which (122.4 gr) takes place at home. Outside the homes, the intake in traditional restaurants (14.3 gr) is much higher than the value obtained from other types of food establishments, which is in consonance with the habitual use of this spice in traditional cuisine [2,24].

### 3.2. Comparison Between the Total Intake of PAHs from Smoked Paprika and the PAH Levels That Are Deemed as Toxic

Table 4 shows the results of the total annual intake per person of B(a)P and PAH4 from the consumption of all kinds of processed meat products, cooked products and seasoned products containing smoked paprika in Extremadura. The calculations were performed with the average levels of B(a)P and PAH4 determined in samples of smoked paprika (POD “Pimentón de La Vera” smoked paprika), as described in the methodology section.

As Table 4 shows, the annual intake of B(a)P and PAH4 from the consumption of smoked paprika is very reduced, which is the consequence of the low amount of smoked paprika being consumed per person per year. The consumption values obtained in this study, however, are in line with the findings of [26] for the subgroup “spices and flavourings”, which show the high consumption level of smoked paprika in Extremadura, again proving the appropriateness of carrying out this study in this region. Despite previous research having found that smoked paprika has the highest PAH content compared to other foods, such as bread and cheese [22], the reduced amount that is consumed per person results in a low annual PAH intake.

Table 5 is a comparison between the data relating to the annual intake of B(a)P and PAH4 on account of smoked paprika consumption, and the annual toxicity levels established according to the recommendations of [12]. Thus, 70 µg per kg of body weight per day was taken as a reference for B(a)P and 340 µg per kg of body weight per day for PAH4. According to [12], the amounts based on kg of body weight were converted into intake per person considering an individual of 70 kg in body weight.

The significant difference presented in Table 5 between the smoked paprika PAH figures and the allowed level figures proves the reduced impact in toxicity terms that the consumption of smoked paprika has in comparison to the toxicity levels allowed for B(a)P and PAH4, in spite of the effect that traditional paprika smoke-drying methods have on the content of polycyclic aromatic hydrocarbons.

The results found in this study are in line with those of the scarce studies on the analysis of PAHs in smoked paprika and in dry red pepper or meat products containing this spice [17,18,19,20,21]. The overall conclusion regarding the levels of PAHs in smoked paprika, as found in the present study, is that they are well below the limits established by law.

Finally, Table 6 presents a complementary approach, as it shows—according to data extracted from [12]—the consumption of B(a)P and PAH4 derived from various categories of food products. With the purpose of allowing a comparison between the categories, information was also added on the respective amounts consumed, except for smoked paprika, given that, as mentioned repeatedly, its consumption is spread over a large variety of food products, which may be cooked (at home or in restaurants) or industrially processed (meat sausages). Therefore, including a value not to represent the consumption of smoked paprika but the amount of food products consumed containing smoked paprika would have been a source of confusion for the reader.

The PAH intake per person was calculated for each category of food products, but these should not be added up in order to estimate a global figure, as one person might not be a consumer of all the food product groups [12]. Sea food and sea products were the main contribution of PAH4 in food, followed by cereals and cereal products, and by the vegetable, pulses and nuts group. Within the large variety of food products containing PAH, the food products contributing the highest levels in the diet are fats, fish and fish products, which is in line with the findings of [11], where the highest levels of PAH are related to the smoking, drying or high-temperature cooking processes, as is the case, for example, of cooking fats and smoked/dried fish.

On the other hand, the Codex Alimentarius [35] established that the food products that contribute the most to the intake of PAHs are cereals and cereal products because they are a frequent item in our diets. In addition to that, vegetable fat and oils also provide an important contribution due to the high levels of PAHs they contain. However, fish and smoked meats and food cooked on the grill, in spite of their high PAH concentrations, do not make a significant contribution, since they are generally a small part of our diets. Particularly, if these food products were habitual in our diet, they would contribute notably to the intake of PAHs and, therefore, would pose a considerable risk to consumer health [35].

The results for the annual consumption of PAH4 from smoked paprika in Extremadura are similar to the data provided for the consumption of sea food and sea products, with the latter group of food products being the one that most contributes to PAH4 intake [35]. This is an interesting ratio because molluscs and, especially, mussels are among the species that are mostly employed as sentinel organisms to detect environmental damage or stress in water ecosystems as they can be found in abundance, have the appropriate size and provide filtering activity [36]. However, the levels of PAH4 consumed are within the maximum levels established by the EFSA. Additionally, in the particular case of B(a)P levels, in smoked paprika, these represent half the amount of sea products.

In terms of the consumption of PAH4 from fish and fish products, the amounts consumed are notably reduced, and this is due to two main reasons, i.e., the first one is that fish has a mechanism that allows it to discharge PAH4 from their system [37], and the second, although not less interesting, is that many of the fish products being consumed are smoked. This could lead us to believe that there are large amounts of these residues derived from smoking techniques, but this is in fact untrue, as since the middle of the 20th century, a relatively new alternative to traditional smoke-drying has been used, i.e., smoke aromas which are commercially produced. These are generic smoke extracts that are filtered and separated from the resin material containing the majority of PAHs [38], which explains the lack of PAHs in the smoked fish samples.

It could, therefore, be considered that the use of synthetic products might be a solution to reduce PAH levels in final products. However, this disregards the consumer, for whom a product to which additives have been added instead of using natural smoking is often not acceptable. This is even more relevant in the case of a traditional product protected by a Denomination of Origin, as is the case of “Pimentón de la Vera” smoked paprika, where the traditional production process plays a major role in the consumer’s perception of the product [39]. It is worth highlighting that among the differential aspects of “Pimentón de La Vera” smoked paprika is its natural character, which sets high expectations for consumers in terms of the quality of the product and its effects on health, which is also the case with other natural spices and flavourings [14,40]. Nevertheless, in a context where the consumption of spices and flavourings is continuously increasing and given that smoked paprika is among the most commonly used products in this category [41], it is necessary for the consumer to be informed of its reduced contribution to PAH intake.

## 4. Conclusions

“Pimentón de La Vera” smoked paprika is a spice produced following traditional procedures, which provides it with distinctive organoleptic characteristics that are highly appreciated by consumers. However, the actual manual techniques employed in the production of the product give rise to undesirable compounds, such as PAHs, and the levels that are allowed to reach consumers have been reduced in recent years due to their effects on health.

This paper is a first approach to the study of the consumption of smoked paprika per person in Spain, with the results showing that the average amount being consumed is 139 g per person per year. According to the average levels of PAHs found in this product, the annual intake of BaP and PAH4 from smoked paprika consumption is, respectively, 7 and 108 µg per person per year. Therefore, its contribution to the intake of PAH is much lower than the amount contributed by other types of food products and, therefore, has a very low impact on the total intake of these substances.

These results help support the use of traditional smoked paprika firewood production processes and could be the basis for any future research intending to reduce the presence of non-desirable compound levels, such as PAHs, without diminishing the sensory traits of smoked paprika. Furthermore, they can contribute to the consolidation and development of the paprika sector, which is among the cornerstones of agribusiness in this region.

## Figures and Tables

**Table 1:** Details on the content of smoked paprika (%) in the various processed meat products produced by 10 Extremaduran companies.

	Meat Products Manufacturer												
	01	02	03	04	05	06	07	08	09	10	Average Smoked Paprika Content in Fresh Products	Average Smoked Paprika Content in Product Ready for Market	SD *
Sausage	1.57	1.50	1.52	1.50	1.55	1.55	1.62	1.51	1.50	1.50	1.53	1.995	0.040
Loin	0.75	0.75	0.78	0.80	0.77	0.77	0.80	0.75	0.78	0.77	0.77	1.001	0.187

* Standard deviation.

**Table 2:** Average consumption of smoked paprika in households and restaurants derived from the consumption of processed meat products and food cooked using smoked paprika.

	“Chorizo”	Dry-Cured Pork Loin	Smoked Paprika Used for Cooking
kg of processed meat products and cooked products consumed per person per year in the households	2.8604	0.9522	0.0558
kg of processed meat products and cooked products consumed per person per year in traditional restaurants	0.3978	0.0824	0.0055
kg of processed meat products and cooked products consumed per person per year in pizza and burger bars and others	0.0337	0.0551	0.0011
Total consumption (kg) of processed meat products and cooked products per person per year	3.2919	1.0897	0.0624
% of smoked paprika in mature product ready for the market	1.9950	1.0010	100.0000
kg of smoked paprika per person per year	0.0657	0.0109	0.0624

Data were collected from surveys distributed to 200 consumers, 40 restaurants and 10 processed meat product companies, following the process explained in the methodology section.

**Table 3:** Total intake per person of smoked paprika in Extremadura in households and restaurants (g person ^−1^ year^−1^).

Smoked Paprika Used in:	“Chorizo”	Dry-Cured Pork Loin	Cooking	Total
Households	57.06	9.53	55.80	122.4
Traditional restaurants	7.94	0.82	5.50	14.3
Pizza, burger and other bars	0.67	0.55	1.10	2.3
Total	65. 7	10.9	62.4	139

Data were collected from surveys distributed to 200 consumers, 40 restaurants and 10 processed meat product companies, following the process explained in the methodology section.

**Table 4:** Annual intake per person of PAHs (µg y^-1^) in Extremadura from the consumption of all kinds of processed meat products, cooked products and products seasoned with smoked paprika.

Total Consumption of Smoked Paprika (g Person^−1^ Year^−1^)	B(a)P ^1^ Level µg kg^−1^	PAH4 ^1^ Level µg kg^−1^	Annual Intake of B(a)P µg Person^−1^ Year^−1^	Annual Intake of PAH4 µg Person^−1^ Year^−1^
139	49	780	7	108

^1^ Average value drawn from the analysis of 144 samples [30]. PAH4: benz(a)anthracene (B(a)A), chrysene (CHR), benzo(b)fluoranthene (B(b)F) and benzo(a)pyrene (B(a)P).

**Table 5:** Comparison between the levels of annual intake of PAHs from consumption of smoked paprika against the annual toxicity levels established according to European Food Safety Authority (EFSA)’s recommendations.

Total Consumption of Smoked Paprika (g Person^−1^ Year^−1^)	Annual Intake of B(a)P (µg Person^−1^ Year^-1^)	Annual B(a)P Toxicity Level (µg Person^−1^ Year^−1^) ^2^	Annual Intake of PAH4 (µg Person^−1^ Year^−1^)	Annual PAHs Toxicity Level (µg Person^−1^ Year^−1^) ^2^
139	7	1.8 × 10^6^	108	8.7 × 10^6^

^2^ Annual toxicity levels for B(a)P and PAH4 are those indicated by EFSA in “The scientific opinion of the panel on contaminants in the food chanel” [11].

**Table 6:** Consumer exposure to B(a)P and PAH4 for each food category for which occurrence data are available. (* The average consumption mean value as reported by 16 Member States only from consumers was calculated [12]; ** data corresponding to consumption in Extremadura.)

Products	Average Consumption (kg Person^−1^ Year^−1^)	Intake of B(a)P (µg Person^−1^ Year^−1^)	Intake of PAH4 (µg Person^−1^ Year^−1^)
* Cereals and cereal products	93.805	25	94
* Sugar and sugar products including chocolate	15.695	2	9
* Fats (vegetable and animal)	13.870	10	65
* Vegetables, nuts and pulses	70.810	18	81
* Fruit	55.845	2	27
* Coffee, tea, cocoa (expressed as liquid)	219.365	8	39
* Alcoholic beverages	150.745	2	9
* Meat and meat products and substitutes	48.180	15	71
* Seafood and seafood products	9.855	13	106
* Fish and fish products	14.965	8	62
* Cheese	15.330	2	7
* Other products		39	185
** Processed and cooked with smoked paprika in Extremadura		7	108

## Data Availability

The data presented in this study are available on request from the corresponding author.

## Supplementary Materials

The following are available online at https://www.mdpi.com/article/10.3390/foods10050973/s1, Table S1: Model of Questionnaire for Meat Manufacturing Companies in Extremadura, Table S2: Consumption of Smoked Paprika Questionnaire (home), Table S3: Consumption of Smoked Paprika Questionnaire (Restaurants), Table S4: Additional information about the samples using for the establishment of the reference values of HAPs in smoke paprika, Table S5: Additional information about the extraction and analytical methods [29] applied for HAPs determination in smoked paprika.

## References

[1] (2007). European Commission Commission Regulation (EC) No 982/2007 registering certain names in the Register of protected designations of origin and protected geographical indications (Pimentón de la Vera (PDO)—Karlovarský suchar (PGI)—Riso di Baraggia biellese e vercellese (PDO)). Off. J. Eur. Union..

[2] Bartolomé T., Coleto J.M., Velázquez R. (2016). Historias de las plantas II: La historia del pimiento. La Agricultura y la Ganadería Extremeñas.

[3] Picón J., Guerra M.L., Garzón C., Sánchez M.C., Simón P., Cepeda N. (2019). Las macromagnitudes agrarias. La Agricultura y la Ganadería Extremeñas en 2018.

[4] Hernández A., Martín A., Aranda E., Bartolomé T., Córdoba M.D.G. (2006). Detection of smoked paprika “Pimentón de La Vera” adulteration by free zone capillary electrophoresis (FZCE). J. Agric. Food Chem..

[5] Hernández A., Martín A., Aranda E., Bartolomé T., Córdoba M.D.G. (2007). Application of temperature-induced phase partition of proteins for the detection of smoked paprika adulteration by free zone capillary electrophoresis (FZCE). Food Chem..

[6] Hernández A., Aranda E., Martín A., Benito M.J., Bartolomé T., Córdoba M.D.G. (2010). Efficiency of DNA typing methods for detection of smoked paprika “pimenton de la Vera” adulteration used in the elaboration of dry-cured iberian pork sausages. J. Agric. Food Chem..

[7] Velázquez R., Casquete R., Hernández A., Martín A., Córdoba M.G., Coleto J.M., Bartolomé T. (2019). Effect of plant density and harvesting type on yield and quality of fresh and dried peppers and paprika. J. Sci. Food Agric..

[8] Singh L., Varshney J.G., Agarwal T. (2016). Polycyclic aromatic hydrocarbons’ formation and occurrence in processed food. Food Chem..

[9] Hokkanen M., Luhtasela U., Kostamo P., Ritvanen T., Peltonen K., Jestoi M. (2018). Critical Effects of Smoking Parameters on the Levels of Polycyclic Aromatic Hydrocarbons in Traditionally Smoked Fish and Meat Products in Finland. J. Chem..

[10] (2006). European Commission Commission Regulation (EC) No 1881/2006 of 19 December 2006 setting maximum levels for certain contaminants in foodstuffs. Off. J. Eur. Union..

[11] Scientific Committee on Food Opinion of the Scientific Committee on Food on the risks to human health of Polycyclic Aromatic Hydrocarbons in Food SCF/CS/CNTM/PAH/29 ADD1. https://ec.europa.eu/food/sites/food/files/safety/docs/sci-com_scf_out153_en.pdf.

[12] European Food Safety Authority Polyciclic Aromatic Hidrocarbons in Food (2008). Scientific opinion of the panel on contaminants in the food chain. EFSA J..

[13] (2015). European Commission Commission Regulation (EU) 2015/1933 of 27 October 2015 amending Regulation (EC) No 1881/2006 as regards maximum levels for polycyclic aromatic hydrocarbons in cocoa fibre, banana chips, food supplements, dried herbs and dried spices. Off. J. Eur. Union..

[14] Schaarschmidt S. (2016). Public and private standards for dried culinary herbs and spices-Part I: Standards defining the physical and chemical product quality and safety. Food Control..

[15] (2014). European Commission Commission Regulation (EU) No 1327/2014 of 12 December 2014 amending Regulation (EC) No 1881/2006 as regards maximum levels of polycyclic aromatic hydrocarbons (PAHs) in traditionally smoked meat and meat products and traditionally smoked fish and fishery. Off. J. Eur. Union..

[16] (2020). European Commission Commission Regulation (EU) 2020/1255 of 7 September 2020 amending Regulation (EC) No 1881/2006 as regards maximum levels of polycyclic aromatic hydrocarbons (PAHs) in traditionally smoked meat and smoked meat products and traditionally smoked fish and smo. Off. J. Eur. Union..

[17] Ishizaki A., Saito K., Hanioka N., Narimatsu S., Kataoka H. (2010). Determination of polycyclic aromatic hydrocarbons in food samples by automated on-line in-tube solid-phase microextraction coupled with high-performance liquid chromatography-fluorescence detection. J. Chromatogr. A.

[18] Rozentale I., Yan Lun A., Zacs D., Bartkevics V. (2018). The occurrence of polycyclic aromatic hydrocarbons in dried herbs and spices. Food Control..

[19] Monago-Maraña O., Pérez R.L., Escandar G.M., Muñoz De La Peña A., Galeano-Díaz T. (2016). Combination of Liquid Chromatography with Multivariate Curve Resolution-Alternating Least-Squares (MCR-ALS) in the Quantitation of Polycyclic Aromatic Hydrocarbons Present in Paprika Samples. J. Agric. Food Chem..

[20] Lorenzo J.M., Purriños L., Bermudez R., Cobas N., Figueiredo M., García Fontán M.C. (2011). Polycyclic aromatic hydrocarbons (PAHs) in two Spanish traditional smoked sausage varieties: “Chorizo gallego” and “Chorizo de cebolla”. Meat Sci..

[21] García-Falcón M.S., Simal-Gándara J. (2005). Polycyclic aromatic hydrocarbons in smoke from different woods and their transfer during traditional smoking into chorizo sausages with collagen and tripe casing. Food Addit. Contam..

[22] Fasano E., Yebra-Pimentel I., Martínez-Carballo E., Simal-Gándara J. (2016). Profiling, distribution and levels of carcinogenic polycyclic aromatic hydrocarbons in traditional smoked plant and animal foods. Food Control..

[23] Martín A., Hernández A., Aranda E., Casquete R., Velázquez R., Bartolomé T., Córdoba M.G. (2017). Impact of volatile composition on the sensorial attributes of dried paprikas. Food Res. Int..

[24] Velázquez R., Hernández A., Martín A., Aranda E., Gallardo G., Bartolomé T., Córdoba M.G. (2014). Quality assessment of commercial paprikas. Int. J. Food Sci. Technol..

[25] Pereira C., Córdoba M.D.G., Aranda E., Hernández A., Velázquez R., Bartolomé T., Martín A. (2019). Type of paprika as a critical quality factor in Iberian chorizo sausage manufacture. CYTA J. Food.

[26] (2020). MAPA Informe del Consumo de Alimentación en España 2019.

[27] Campillo J.E., Tormo M.A., Gómez-Encinas J., Campillo C., Viñas J., Borrás C., Torres M.D. (2010). Antioxidant andhypolipidemic effect of smoked paprika in healthy subjects. CyTA J. Food.

[28] Tormo M.A., Campillo J.E., Viñas J., Gómez-Encinas J., Borrás C., Torres M.D., Campillo J.E. (2013). The mechanism of the antioxidant effect of smoked paprika from La Vera, Spain. CyTA J. Food.

[29] MAPA Observatorio del Consumo y la Distribución Alimentaria. https://www.mapa.gob.es/es/alimentacion/temas/consumo-tendencias/observatorio-de-consumo-y-la-distribucion-alimentaria/.

[30] (2019). Universidad de Extremadura Informe del contenido de hidrocarburos policíclicos aromáticos en pimentón con DOP “Pimentón de La Vera”, correspondientes a las campañas 2015 a 2018. Laboratorio agroalimentario de la Escuela de Ingenierías Agrarias; Badajoz, Spain.

[31] Pule B.O., Mmualefe L.C., Torto N. (2012). Analysis of polycyclic aromatic hydrocarbons in fish with Agilent bond elut QuEChERS AOAC kit and HPLC-FLD. Application Note. Proven Approaches for Today’s FOOD analysis Challenges.

[32] Embutidos Rodríguez Como Hacer Chorizo Casero en Diez Sencillos Pasos. https://embutidosrodriguez.es/como-hacer-chorizo-casero-en-10-sencillos-pasos/.

[33] Centro de Investigación y Tecnología Agroalimentaria de Aragón Calidad de Canal, Carne y sus Derivados Apreciación Visual de Chorizos: Resultados. https://calidadcarnecita.wordpress.com/2016/03/15/apreciacion-visual-de-chorizos-resultados/.

[34] Martín V.J. (2012). Consumo de embutidos y salazones en España. Distrib. y Consum..

[35] FAO-WHO (2009). Code of Practice for the Reduction of Contamination of Food with Polycyclic Aromatic Hydrocarbons (PAH) from Smoking and Direct Drying Processes.

[36] Perez-Cadahia B., Laffon B., Pasaro E., Mendez J. (2004). Evaluation of PAH bioaccumulation and DNA damage in mussels (Mytilus galloprovincialis) exposed to spilled Prestige crude oil. Comp. Biochem. Physiol. Part. C Toxicol. Pharmacol..

[37] Law R.J., Kelly C., Baker K., Jones J., McIntosh A.D., Moffat C.F. (2002). Toxic equivalency factors for PAH and their applicability in shellfish pollution monitoring studies©British Crown copyright 2002. J. Environ. Monit..

[38] Stolyhwo A., Sikorski Z.E. (2005). Polycyclic aromatic hydrocarbons in smoked fish—A critical review. Food Chem..

[39] Mesías F.J., Gaspar P., Escribano M., Pulido F. (2010). The role of protected designation of origin in consumer preference for iberian dry-cured ham in Spain. Ital. J. Food Sci..

[40] Embuscado M.E. (2015). Herbs and spices as antioxidants for food preservation. Handbook of Antioxidants for Food Preservation.

[41] Topuz A., Dincer C., Özdemir K.S., Feng H., Kushad M. (2011). Influence of different drying methods on carotenoids and capsaicinoids of paprika (Cv.; Jalapeno). Food Chem..

