# Supplementary material for: An Approach to the Consumption of Smoked Paprika in Spain and Its Impact on the Intake of Polycyclic Aromatic Hydrocarbons
Source: Foods. 2021 Apr 29;10(5):973. doi: 10.3390/foods10050973 (PMC8145680; doi:10.3390/foods10050973)
Supplement: Supplementary file 1 [file foods-10-00973-s001.zip › foods-1165177-supplementary.pdf]

foods MDPI

Supplementary materials:

Table S1. Model of Questionnaire for Meat Manufacturing Companies in Extremadura

Company No.	Municipality	Red Chorizos and Sliced Versions	Pork Loins, Similar Meats and Their Sliced Versions
% of paprika in industrial manufactured fresh meat			
% of weight reduction of the average industrial matured meat ready for the market			

Table S2. Consumption of Smoked Paprika Questionnaire (home)

Introductory Questions		
Group of municipalities		Municipality
Job of the Chief Income Earner		Work status
No. of family members >6 years old in the household		Age of the person in charge of the grocery shopping
**Questions about the consumption at home**		
**Product description**	**Frequency**	**Notes**
2.1. Iberian smoked-flavour red traditional *chorizo* or *morcón* (800 g)	Not at all<br>< 1/2 *chorizo* a week (please specify)<br>1/2 *chorizo* a week<br>> ½ *chorizo* a week (please specify)	
2.2. Iberian smoked-flavour red horseshoe-shaped *chorizo* (350 g)	Not all at<br>< 1/2 *chorizo* a week (please specify)<br>1/2 *chorizo* a week<br>> ½ *chorizo* a week (please specify)	
2.3. Iberian red pork loin (1,100 g)	Not at all<br>1 pork loin a year<br>2 pork loins a year<br>> 2 pork loins a year (please specify)	
2.4. Red shoulder blade (Iberian pork butt) (400 g)	Not at all<br>1 pork loin a year<br>2 pork loins a year<br>> 2 pork loins a year (please specify)	
2.5. Sliced Iberian smoked-flavour red traditional *chorizo* or *morcón* (100 g)	Not at all<br>< 1/2 packet a week (please specify)<br>1/2 packet a week<br>> ½ packet a week (please specify)	
	Not at all	

2.6. Sliced Iberian red pork loin or shoulder blade (100 g)	< 1/2 packet a week (please specify)<br>1/2 packet a week<br>> ½ packet a week (please specify)
2.7. Smoke-dried paprika tin (70 g?) used as food seasoning	Not at all<br>< 1 tin a year (please specify)<br>1 tin a year<br>> 1 tin a year (please specify)
2.8. Details on the making of marinated meats	Grams of smoked paprika per kilogram of marinated product<br>% of paprika on the total employed for the marinate that is present in the juice that is not used<br>% of the paprika purchased in packets that is dedicated to food marinates

Eating out

**Description**	**Frequency**	**Type of establishment (B= Burger bars; P= Pizza restaurants; W= Wok restaurants; CH= Chinese restaurants; RT= Traditional restaurants)**					
		**Total**	**B**	**P**	**Other W**	**Other CH**	**RT**
3.1. Eating out – all members of the household	Never or less than once a month (please specify)<br>Once a month<br>Twice a month<br>> Twice a month (please specify)						
3.2. Eating out – 1 member of the household	Never or less than once a month (please specify)<br>Once a month<br>Twice a month<br>> Twice a month (please specify)						
3.3. Eating out – 2 members of the household	Never or less than once a month (please specify)<br>Once a month<br>Twice a month<br>> Twice a month (please specify)						
3.4. Eating out – 3 members of the household	Never or less than once a month (please specify)<br>Once a month<br>Twice a month<br>> Twice a month (please specify)						
	Never or less than once a month (please specify)						

3.5. Eating out – 4 members of the household	Once a month<br>Twice a month<br>> Twice a month (please specify)
3.6. Eating out – 5 members of the household	Never or less than once a month (please specify)<br>Once a month<br>Twice a month<br>> Twice a month (please specify)
3.7. Eating out – 6 members of the household	Never or less than once a month (please specify)<br>Once a month<br>Twice a month<br>> Twice a month (please specify)

Table S3. Consumption of Smoked Paprika Questionnaire (Restaurants).

**Introductory Questions**		
Group of municipalities		Municipality
Type of establishment	Type of establishment (B= Burger bars; P= Pizza restaurants; W= Wok restaurants; CH= Chinese restaurants; RT= Traditional restaurants)	
**Questions about consumption at the specified frequency**		
2.1. Smoked-flavour red traditional *chorizo* or *morcón* (800 g)	Not at all<br>< 1/2 *chorizo* a week (please specify)<br>1/2 *chorizo* a week<br>> ½ *chorizo* a week (please specify)	
2.2. Smoked-flavour red horseshoe-shaped *chorizo* (350 g)	Not all at<br>< 1/2 *chorizo* a week (please specify)<br>1/2 *chorizo* a week<br>> ½ *chorizo* a week (please specify)	
2.3. Iberian red pork loin (1,100 g)	Not at all<br>1 pork loin a year<br>2 pork loins a year<br>> 2 pork loins a year (please specify)	
2.4. Red shoulder blade (Iberian pork butt) (400 g)	Not at all<br>1 pork loin a year<br>2 pork loins a year<br>> 2 pork loins a year (please specify)	
2.5. Sliced Iberian smoked-flavour red traditional *chorizo* or *morcón* (100 g)	Not at all<br>< 1/2 packet a week (please specify)<br>1/2 packet a week<br>> ½ packet a week (please specify)	

2.6. Sliced Iberian red pork loin or shoulder blade (100 g)	Not at all<br>< 1/2 packet a week (please specify)<br>1/2 packet a week<br>> ½ packet a week (please specify)
2.7. Smoke-dried paprika tin (70 g?) used as food seasoning	Not at all<br>< 1 tin a year (please specify)<br>1 tin a year<br>> 1 tin a year (please specify)
2.8. Details on the making of marinated meats	Grams of smoked paprika per kilogram of marinated product<br>% of paprika on the total employed for the marinate that is present in the juice that is not used<br>% of the paprika purchased in packets that is dedicated to food marinates

3. Average no. of meals served at the specified frequency (week, moth, year)

Table S4. Additional information about the samples using for the establishment of the reference values of HAPs in smoke paprika.

**Analysis of Smoked Paprika (sampling)**				**Descriptive Statistics**	**PAHs[2]**	
Season		Processing type				
Year	N	Type[1]	N		B(a)P	PAH4
2015	41	Type 1	53	Mean	49	780
2016	33	Type 2	17	Standard Deviation	36	485
2017	37	Type 3	74	Minimum	12	101
2018	33			Maximum	192	1817
Total	144	Total	144			

[1] Differences of the processing are related to drying temperature, bonfire location and application of forced air.

[2]Values of PAHs in µg kg-1.

Table S5. Additional information about the extraction and analytical methods [29] applied for HAPs determination in smoked paprika.

**HAPs**	**QuEChERS[1]**		**HPLC-FLD[2]**	
	**% Recovery**	**%RDS**	**LOD**	**LOQ**
Benzo(a)anthracene (B(a)A)	94.2	1.7	0.82	2.73
Chrysene (CHR)	98.7	1.2	2.80	9.32
Benzo(b)fluoranthene (B(b)F)	95.4	1.5	0.60	2.00
Benzo(a)pyrene (B(a)P)	93.2	1.9	0.08	0.27

[1]N=10

[2] Excitation 260 nm and emission 240 nm wavelengths. Limit of detection (LOD) and limit of quantification (LOQ).
